# The effect of the Mediterranean diet combined with physical activity on cognitive function in older adults: a scoping review

**DOI:** 10.1080/15502783.2026.2696421

**Published:** 2026-07-22

**Authors:** Yi Yang, Rui Du, Changhong Wu, Shuixiong Liao, Tingran Zhang, Jiong Luo

**Affiliations:** a Research Centre for Exercise Detoxification, College of Physical Education, Southwest University, Chongqing, China; b Chongqing Beibei Vocational High School, Chongqing, China; c Dongguan University of Technology, Dongguan, China

**Keywords:** Physical activity, mediterranean diet, cognitive function, care, oxidative stress

## Abstract

**Background:**

Cognitive impairment and dementia are rapidly increasing among older adults, with limited pharmacological treatments available. Modifiable lifestyle factors, particularly diet and physical activity, are promising preventive targets. Although the Mediterranean diet and regular exercise individually benefit cognition, their combined effects and underlying mechanisms remain poorly synthesised.

**Methods:**

The systematic review followed PRISMA-ScR guidelines and involved database searches on PubMed, Embase, Web of Science, Cochrane, and CNKI. Study inclusion criteria were defined using the PICO framework.

**Results:**

The Mediterranean diet supplies polyphenols and fatty acids that reduce oxidative stress and inflammation, and support neurotrophic and neuroendocrine functions, thus protecting the brain. Meanwhile, physical activity boosts cerebral blood flow, improving antioxidant delivery and stimulating neurotrophic factors like BDNF, IGF-1, and VEGF to enhance brain plasticity. Together, these approaches act synergistically to balance energy metabolism, lower inflammation, and activate antioxidant defenses, thereby preserving brain homeostasis and boosting cognitive function.

**Conclusions:**

Evidence suggests a synergistic effect of the Mediterranean diet and physical activity in preserving cognitive function in older adults. The diet directly supports cognitive health and provides essential nutrients for exercise-induced neuroregeneration. Physical activity, in turn, increases cerebral blood flow, improving nutrient delivery and stimulating neurotransmitter release. This interaction enhances brain plasticity, establishing a virtuous cycle between nutrition, exercise, and cognitive health.

## 1. Introduction

According to the World Health Organization's 2025 Global Report on Cognitive Health, the prevalence of dementia among adults aged 80 and over is 40%, with associated socio-economic costs projected to exceed US$3 trillion by 2050 [1]. Dementia is a leading cause of health impairment and reduced life expectancy in older adults [2]. Furthermore, cognitive impairment accompanied by severe behavioral abnormalities has become a major threat to the quality of life of older adults, ranking after cardiovascular diseases and malignant tumors [3]. Consequently, identifying strategies to delay cognitive decline has become a major focus of international basic and clinical research. Physical activity (PA) and dietary intake are the two primary controllable factors influencing health in older adults [4]. Research demonstrates that PA level is a significant predictor of cognitive decline [5,6]. Conversely, unhealthy dietary habits can contribute to cognitive impairment in this population [3,7]. Studies on the relationship between diet and cognitive function suggest that consuming foods high in folate, such as spinach and pig liver, may help delay cognitive decline [8]. Another important molecule is the antioxidant carotenoid astaxanthin, which is abundant in aquatic animals like prawns, salmon, and lobsters. Astaxanthin can cross the blood-brain barrier, exerting antioxidant, anti-inflammatory, and neuroprotective effects on the central nervous system [9]. In contrast, dietary patterns high in pickled foods and meat products are associated with a significantly elevated risk of cognitive impairment [10]. This evidence underscores the critical role of diet in promoting healthy cognitive ageing.

The Mediterranean Diet (MD) was first proposed by nutritionist Ancel Keys in 1960 [11], initially defined as a dietary pattern low in saturated fats and high in plant oils. MD is a term used to describe the traditional eating habits of people in Crete, Southern Italy, and other Mediterranean countries [12]. Guided by the food pyramid, the MD centers on relative proportions and frequency of consumption across food groups rather than absolute quantities. This model is plant-based, with olive oil as the primary fat source [13]. Animal-based foods follow the principle of prioritizing fish, moderate poultry and eggs, and limiting red meat. Additionally, it incorporates a sociocultural dimension, advocating shared meals, regular physical activity, and seasonal eating, thereby forming a holistic healthy lifestyle pattern [14]. It has now become one of the most extensively studied and highly regarded dietary patterns worldwide. Originating from the traditional eating habits of Southern Europe, it is characterized by a balance of plant-based foods and healthy fats [15–18]. Its principal components include a plant-based foundation of vegetables, fruits, whole grains, legumes, and nuts, which provide abundant dietary fiber and antioxidants. Extra virgin olive oil, rich in monounsaturated fatty acids (MUFA) and anti-inflammatory compounds, serves as the primary fat source. Fish, such as salmon and sardines, are recommended two to three times per week to supply omega-3 fatty acids. Dairy consumption should be moderate, prioritizing fermented products like yoghurt and cheese, while egg intake is typically limited to no more than one per day. The diet restricts red meat to occasional, small portions and minimizes the intake of processed foods and refined sugars. Beyond food composition, the MD emphasizes a supportive culinary culture that includes social dining, mindful eating, and, for those who consume alcohol, moderate red wine intake (generally defined as up to two glasses daily for men and one for women, consumed with meals).

Extensive research has confirmed that this dietary pattern is associated with numerous health benefits, including a reduced incidence of type 2 diabetes, obesity, metabolic syndrome, depression, cognitive decline, and kidney stones [15,17–19]. Notably, its role in promoting cognitive function in older adults has received significant attention. Adherence to the traditional MD, characterized by high consumption of seafood, fruits, and nuts, is associated with a significant reduction in dementia risk of up to 23% [20]. These cognitive benefits are observed irrespective of existing cognitive impairment, with both long- and short-term adherence conferring positive effects, particularly on memory [21]. Furthermore, a dose-response relationship exists, whereby greater adherence to the diet correlates with more pronounced improvements in overall cognitive function in older adults [21]. The diet's neuroprotective effects are attributed to its rich composition of whole grains, fruits, vegetables, monounsaturated fatty acids from extra virgin olive oil, fish, and seafood, which are known to delay cognitive decline and reduce the risk of Alzheimer's disease (AD) [22]. Importantly, the MD is part of a broader lifestyle paradigm that integrates dietary patterns with regular PA and social engagement [14,23]. For instance, research suggests that combining aerobic exercise with the MD can enhance cognitive function in postmenopausal women and may mitigate the adverse effects of sex hormone deficiency on brain health [23]. Similarly, consuming foods rich in vitamin D, such as salmon and cheese, combined with adequate outdoor sun exposure, may help prevent cognitive impairment in older adults [24].

Combining PA with an MD may produce synergistic benefits for cognitive function in older adults, although the underlying mechanisms remain unclear. This paper investigates the effects and mechanisms of these interventions to inform the development of strategies for mitigating cognitive decline and enhancing quality of life in older adults.

## 2. Methods

Scoping reviews are a systematic methodology for knowledge synthesis that maps the trajectory of a body of literature by retrieving, screening, and synthesizing existing evidence. They serve to clarify key disciplinary concepts, identify research trends, demonstrate the breadth of existing knowledge, and outline directions for future inquiry [25]. This study was conducted in strict accordance with the PRISMA-ScR (Preferred Reporting Items for Systematic Reviews and Meta-Analyses—Extension for Scoping Reviews) guidelines established by Tricco et al. [26].

### 2.1. Literature search strategy

Two researchers conducted a systematic literature search across five databases: PubMed, Web of Science, Embase, Cochrane, and the China National Knowledge Infrastructure (CNKI). This search strategy is based on the PICOS framework, encompassing population (“older adults”), intervention (“Mediterranean diet combined with exercise”), comparison (“no intervention”), outcome (“cognitive function”), and study design (“randomized controlled trials, cross-sectional studies”). Key terms include physical activity, exercise intervention, Mediterranean diet, cognitive function, executive function, inhibitory control, and memory. A combined search strategy of subject headings and free-text terms was employed. The search was limited to literature published from January 2000 to January 2026.

### 2.2. Inclusion and exclusion criteria

The literature screening, inclusion, and exclusion criteria were defined according to the PICOS framework.

The inclusion criteria were: (1) participants aged 60 years or older; (2) studies investigating combined interventions of physical activity and the MD, with all study designs eligible; (3) exercise prescriptions adhering to the American College of Sports Medicine (ACSM) standards; and (4) primary outcomes including cognitive ability, executive function, working memory, or inhibitory control.

The exclusion criteria were: (1) publications not in English or Chinese; (2) literature unrelated to exercise prescription, the MD, older adults, or cognitive function; (3) review articles, theses, conference proceedings, qualitative studies, or publications with inaccessible data; and (4) articles with missing findings or data.

### 2.3. Literature screening

The literature was managed using NoteExpress software to eliminate duplicate records. Two investigators independently screened the records by reviewing titles and abstracts. Articles deemed potentially eligible underwent full-text assessment. The two reviewers were required to reach a consensus on the selections. In cases of disagreement, a third reviewer was consulted for arbitration. Finally, the compiled literature database was cross-checked and consolidated by a third party.

## 3. Results

### 3.1. Inclusion in research evidence

**Figure 1.**
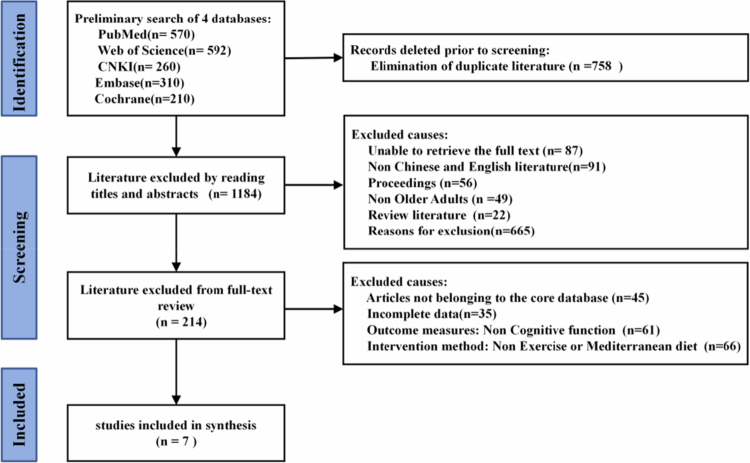
Literature retrieval and screening process.

Through a systematic search of electronic databases, 1942 publications were initially identified. Following the removal of duplicates, 1184 records were screened based on their titles and abstracts. This led to the retrieval of 214 full-text articles for eligibility assessment, of which 204 were excluded for not meeting the inclusion criteria. Consequently, 7 studies were included in the scoping review (Figure 1). The baseline characteristics of the included studies are summarized in Table 1.

**Table 1.** Characteristics of the included studies.

Study details	Country	Age\n\gender	Cycle	Model	Exercise program	Dietary plan	Cognitive measure	Cognitive effect	Research type
[23]	Egypt	65.4 ± 2.8; *N* = 30 (Older Adults); Female (30)	12 weeks	MD+PA	3×/week, 40 min/session, 60%–70% HRmax, running	Low-calorie diet	RUDAS	Cognitive function↑	RCT
[27]	United Kingdom	67.4 ± 4.6; *N* = 104 (Older Adults); Female (77), Male (27)	24 weeks	MD+PA	150 min of MI or 75 min of HIIT per week	MD	NTB test	Global cognitive function↑ Memory↑	RCT
[28]	India	68.3 ± 5.8; *N* = 60 (Older Adults); Female (22), Male (38)	24 weeks	MD+PA	Aerobic and resistance training twice a week	MD, with 20% protein, 25% fat, and 55% carbohydrates	PGI-MS	Cognitive function↑Memory↑	RCT
[29]	Israel	65.00 ± 2.74; *N* = 284 (Older Adults) ; Female (34), Male (250)	72 weeks	MD+PA	20 min\sessions, 5 sessions\week,	Green MD	MRI	No significant change (brain volumes↑)	RCT
[30]	Australia	60–90 years; *N* = 102 (Older Adults) Female (73), Male (29)	24 weeks	MD+PA	Aerobic exercise 30–40 min/day	MD (olive oil as primary fat)	SWM test;	No significant change	RCT
[24]	China	≥60; *N* = 1115 (Older Adults);	–	MD+PA	Outdoor aerobic exercise	MD	MMSE	Cognitive function↑	CS
[31]	America	68.0 ± 10.0; *N* = 3463; Female = 2012, Male = 1451	–	MD+PA	Vigorous PA ≥ 2 times/week	MD	Global Cognition Test	Global cognitive function↑	CS

Note: *N*: total sample size; RUDAS: Rowland Universal Dementia Assessment Scale; MD: Mediterranean diet; PA: physical activity; HIIT: high-intensity activity; MI: moderate intensity; NTB: Neuropsychological Test Battery; SWM: Spatial Working Memory; CVLT-II: the California Verbal Learning Test; MMSE: Mini-Mental State Evaluation; CS: Cross-sectional; ↑: Significantly improved.

### 3.2. Selected studies

The seven studies reviewed in this research span multiple countries, including Egypt [23], the United Kingdom [27], India [28], Israel [29], Australia [30], China [24], and the United States [31], demonstrating the international diversity of the research samples. In terms of study design, five were randomized controlled trials (RCTs) [23,27–30], and two were cross-sectional studies (CS) [24,31]. In the RCTs, intervention durations ranged from 12 weeks [23], 24 weeks [27,28,30], to 72 weeks [29], while the cross-sectional studies did not specify a fixed intervention period [24,31]. All studies employed an “MD combined with physical exercise” intervention model. Exercise regimens in RCTs encompassed moderate-intensity aerobic training [23], combinations of moderate- and high-intensity interval training [27], aerobic plus resistance training [28], and regular aerobic activity [29,30]. Dietary protocols included standard MD [27,28,30,31], a low-calorie MD [23], a green MD [29], and an MD featuring olive oil as the primary fat source [30]. For cognitive function assessment, these studies employed multiple standardized neuropsychological and imaging tools, primarily including: the Rowland Universal Dementia Assessment Scale (RUDAS) [23], Neuropsychological Test Battery (NTB) [27], Post Graduate Institute Memory Scale (PGI-MS) [28], structural magnetic resonance imaging (MRI) [29], Spatial Working Memory Test (SWM) [30], Mini-Mental State Examination (MMSE) [24], and Global Cognition Test [31]. These tools comprehensively evaluated the potential impact of combined MD and exercise interventions of varying intensities and types on cognitive health in older adults across multiple dimensions, including overall cognitive status, specific memory functions, executive abilities, and brain structural morphology.

### 3.3. Effects of physical activity and Mediterranean diet interventions on cognitive function in older adults

#### 3.3.1. The effect of Mediterranean diet intervention on cognitive function in older adults

Since the nutrients necessary for physiological functions are derived from the diet, healthy eating is a critical modifiable factor for health. A fundamental question, therefore, is what constitutes a healthy diet. To answer this, it is essential to understand the role of nutrients in neuronal function and cognitive performance. Research has shown that essential amino acids, fatty acids, vitamins, and minerals are essential to maintain human health [32,33]. Dietary amino acids are primarily obtained from meat, whereas vitamins and minerals are abundant in fruits, vegetables, legumes, and nuts [33]. The brain utilizes these nutrients to support crucial processes, including neuronal survival, nerve impulse transmission, neurotransmitter synthesis, membrane integrity, synaptic plasticity, and metabolic functions such as energy metabolism and homocysteine regulation [34,35]. These mechanisms are integral to brain health and are strongly linked to cognitive function [36,37]. The MD pattern is associated with promoted health and delayed age-related cognitive decline [38–40]. Its core components comprise extra virgin olive oil, a variety of fruits, particularly dark leafy greens, pulses, nuts, whole grains, and moderate amounts of fish and meat [41]. This pattern recommends limiting dairy products and red wine, while strictly controlling the consumption of eggs and sweets. Studies indicate that following a MD intervention, participants demonstrated improved adherence to most dietary components, particularly fish, nuts, and olive oil, while consumption of sugary beverages was already at a low level before the intervention [27]. Excessive intake of saturated fatty acids may contribute to cognitive impairment and increase the risk of Mild Cognitive Impairment (MCI) or dementia [42]. Moreover, the MD not only helps maintain cognitive and physical function levels but also exerts a positive regulatory effect on hormonal changes in postmenopausal women [23]. Rich in vitamins, minerals, and antioxidants, the MD helps maintain vascular and nervous system health, which may delay cognitive decline [43,44]. Furthermore, the flavonoids found in berries, known for their anti-inflammatory and antioxidant effects, are involved in neuronal signalling and accumulate in specific brain regions, thereby promoting learning and memory functions [45]. Nuts and olive oil are rich sources of dietary fatty acids. These fatty acids exhibit anti-inflammatory properties and contribute to vascular health [46]. Dietary polyphenols, also present in these foods, may enhance cognitive function and mitigate dementia risk by supporting neuronal signalling pathways and through their antioxidant and anti-inflammatory effects [28,46]. Evidence indicates that these nutrients significantly improve various cognitive domains in older adults, including executive function, memory, learning capacity, and abstract reasoning [47]. Moreover, this dietary pattern is associated with a reduction in multidimensional cognitive risk, slowing overall cognitive decline, and improving specific functions such as episodic and prospective verbal memory, as well as information processing speed [48,49]. Knight et al. identified the MD as an effective strategy for reducing the risk of age-related cognitive impairments, including subclinical decline, mild cognitive impairment, AD, and dementia in older Western populations [39]. Regarding the timing of these benefits, research with an average 18-month follow-up showed that participants with strict adherence to the MD achieved higher executive function scores on the MMSE and Stroop tests [50]. Research has shown that postmenopausal women who follow a low-calorie MD experience a significant increase in RUDAS score from 24.23 ± 1.10 to 25.07 ± 0.94 after 12 weeks, and their functional independence score also improves significantly [23]. In a cross-sectional study of 1115 elderly Chinese individuals, the group with high intake of vitamin D-rich foods (e.g. sea fish, eggs) had significantly higher MMSE scores than the low intake group; multivariate regression confirmed that high vitamin D food intake is a protective factor for cognitive function [24]. In the MedEx-UK trial, participants who received a 24-week MD intervention alone showed a 3.7-point increase in MD Adherence Screening Scale (MEDAS) scores compared to the control group, and overall cognitive and memory function also improved significantly [27]. Additionally, elderly individuals with subjective cognitive decline who received “cognitive training plus Mediterranean-equivalent diet” exhibited a 12.25% increase in the total score of the PGI memory scale from baseline, outperforming the health awareness control group [28]. Hardman et al. observed that the MD score improved in the single diet group after 6 months, but the primary cognitive outcome did not reach statistical significance, which may be attributable to sample size and compliance [30]. A cross-sectional analysis of 3463 community-dwelling elderly residents showed that those who adhered solely to the MD had an overall cognitive score 0.81 points higher than those who did not adhere, and a 32% lower risk of cognitive decline [31].

#### 3.3.2. The impact of physical activity on cognitive function in older adults

Exercise is an established intervention for mitigating cognitive decline in older adults [51]. Effective modalities include aerobic exercise [52], resistance training [53], multi-modal programs [54], and traditional Chinese exercises such as Tai Chi [55]. Exercise not only boosts positive emotions in older adults but also enhances their spatial working memory abilities [30]. Moderate-intensity running can enhance cognitive and physical function in women, compensating for the deficiency of sex hormones in postmenopausal women and thereby delaying brain aging [23]. Higher PA levels are consistently associated with a reduced risk of cognitive decline and dementia [38]. Aerobic exercise improves cardiovascular health, evidenced by a 7.8% increase in maximal oxygen uptake (VO₂max) [56], and benefits cognitive functions such as spatial memory, correlating with a 2% increase in anterior hippocampal volume and elevated hippocampal brain-derived neurotrophic factor (BDNF) levels [56]. Regular moderate-intensity aerobic exercise (recommended as 30 min on most days) can improve executive function and memory in healthy older adults [57]. Resistance training, both acute and chronic, also demonstrates potential for cognitive improvement [58]. For instance, older adults undergoing acute resistance training perform significantly better on Stroop test measures than controls [59]. Even when outdoor activities are restricted due to the pandemic, maintaining regular aerobic exercise continues to have a positive effect on cognitive function in older adults [27]. Furthermore, moderate-to-high intensity resistance training once or twice weekly for 12 months can enhance cognitive performance [60]. Research on Tai Chi shows it significantly enhances positive emotional memory and increases activation in the left dorsolateral prefrontal cortex (L-DLPFC), underscoring its unique potential as a cognitive-emotional intervention [55]. Research has shown that regular PA has a protective effect on cognitive function in older adults. Elsayed et al. found that after 12 weeks of moderate-intensity aerobic exercise (3 times per week, 40 min per session, at 60%–70% of maximum heart rate), the cognitive function (RUDAS score) and functional level (FIM score) of postmenopausal, obese elderly women significantly improved [23]. Elderly individuals who engaged in 150 min of moderate-intensity or 75 min of high-intensity PA per week for 24 weeks had significantly better overall cognitive and memory functions than the control group [27]. In addition, a 24-week multimodal intervention found that elderly individuals who combined aerobic and resistance exercise (twice per week, with gradually increasing intensity to 80% HRmax or 80% 1RM) showed the most significant improvements in cognitive domains such as PGI-MS total score, mental balance, attention and concentration, immediate recall, and visual retention [28]. Walking exercise (30–40 min per day) improved spatial working memory (SWM) and emotional state (DASS total score) in elderly people [30]. A cross-sectional study of 3463 community-dwelling elderly residents found that those who engaged in high-intensity PA (≥2 times per week) had an overall cognitive score 0.98 points higher than the non-exercise group (*p* < 0.001) and a 31% reduced risk of cognitive decline [31]. A survey of the elderly population in China similarly found that the high outdoor activity group had significantly higher MMSE scores than the low outdoor activity group, indicating that outdoor activity is an independent protective factor for cognitive function [24]. Therefore, exercise remains a viable strategy for alleviating cognitive decline in older adults.

#### 3.3.3. The effect of a Mediterranean diet combined with physical activity intervention on cognitive function in older adults

Both exercise and the MD are recognized as significant modulators in delaying cognitive decline in older adults [61]. Research investigating their combined effect has demonstrated superior benefits. The study demonstrated that, compared to adhering solely to the MD, combining this diet with moderate-intensity running three times per week for three months led to significant improvements in participants' sex hormone levels, cognitive function, and physical performance [23]. The underlying mechanisms involve the modulation of neuroplasticity substrates, such as neurotrophic signalling, neurogenesis, inflammation, stress response, and antioxidant defenses, by both PA and diet, while cognitive engagement further enhances brain and cognitive reserve [62]. The combined intervention effectively improved overall cognitive function and composite memory scores (particularly verbal memory) over 24 weeks, with the most significant improvement observed in MEDAS scores [27]. Multimodal non-pharmacological interventions, including computerized cognitive training, MD programs, and regular exercise have been shown to significantly improve multiple cognitive domains (e.g. memory, executive function, processing speed) in older adults with subjective cognitive impairment [28]. Age-related brain atrophy, a key biomarker of cognitive decline detectable via MRI is also affected by these interventions. For instance, the MD demonstrates a more pronounced protective effect on slowing hippocampal and lateral ventricle volume (LVV) atrophy in older adults compared to conventional healthy dietary guidelines (HDG) [29]. Furthermore, regardless of age, gender, or cardiovascular risk factors, low adherence to the MD was associated with a higher degree of brain atrophy over a three-year period [63]. However, studies show that polyphenol-based interventions yield only modest improvements in brain volume. In contrast, structural plasticity in the ageing brain appears to be primarily driven by sustained, high-intensity exercise [29,56]. Notably, participants in the combined intervention demonstrated confidence (perceived control) and motivation to change their diet and increase physical activity [27]. The combined intervention (MD + PA) has shown superior cognitive protective effects compared to single interventions in multiple studies, exhibiting dose-response and synergistic effects. Elsayed et al. conducted an RCT showing that after 12 weeks, the RUDAS score of the MIND diet plus moderate-intensity running combination group increased from 24.07 ± 1.08 to 26.57 ± 1.17 (*p* < 0.01), and the improvement was significantly greater than that of the MIND diet alone group [23]. A cross-sectional study also found that the overall cognitive score of the PA + MD combined group was 0.98 points higher than that of non-adherents to either intervention, and 0.60 points higher than that of the PA alone group, confirming the “additive effect” of the combined intervention [31]. After 24 weeks, the MD + PA group showed an overall cognitive z-score improvement of 0.22, a memory domain improvement of 0.31, and dietary behavior changes were maintained for 12 months [27]. The group with high intake of vitamin D-rich foods and high outdoor activity had the highest MMSE score, showing a cumulative protective effect [24]. In addition, the total PGI memory score in the third mock examination combination group of “cognitive training + diet + exercise” increased by 17.79% from baseline, significantly better than that in the single cognitive training group (12.25%) and the control group (1.62%) [28]. The high-polyphenol green MD plus physical activity group showed a significantly smaller decrease in hippocampal occupancy score compared to the healthy dietary guidelines group, and also had less lateral ventricular volume expansion, suggesting that combined intervention can delay age-related brain atrophy [29]. In Hardman et al.'s LIILAC study, the combined group showed significantly better spatial working memory performance than the control group, and the DASS emotional total score was also significantly reduced [30]. Therefore, combined interventions integrating exercise with the MD exert a synergistic protective effect on cognitive health.

## 4. Discussion

### 4.1. The effect of a Mediterranean diet combined with physical activity intervention on cognitive function in older adults

Diet and exercise are pivotal in health maintenance and disease prevention or delay. The global population of elderly individuals with age-related cognitive impairment is steadily growing, and the prevalence of dementia is projected to rise sharply by 2050, while effective pharmacological treatments remain limited [1]. As modifiable factors, diet and exercise can influence disease risk and alter pathophysiological mechanisms [4,64]. High adherence to the MD reduces the risk of overall cognitive decline in older adults free of dementia [65]. Specific micronutrients, including polyphenols, omega-3 fatty acids, and B vitamins, are associated with enhanced cognitive function [22]. The impact of the MD on health-related indicators is highly dependent on its associated lifestyle, including but not limited to high-intensity physical activity, social support, and adequate rest [12]. Consequently, regular PA and balanced nutrition are vital components of a healthy lifestyle for preventing cognitive decline and maintaining mental capacity in older adults. Based on an analysis of seven studies, this study found that combining the MD with exercise significantly improved cognitive function in middle-aged, elderly, and people with cognitive decline compared to either intervention alone. The MD is a key factor for cognitive protection, while exercise needs to be combined with diet to exert broader cognitive benefits. These findings are broadly consistent with previous research [4,64]. Multimodal interventions (diet, exercise, and other modalities) yield the greatest effects on specific cognitive domains such as attention, memory, and spatial working memory, and also have a protective effect on brain structures, including hippocampal volume. Furthermore, improvements in dietary behavior are easier to maintain than those in exercise, and early intervention (e.g. around age 50) may be more effective in delaying cognitive decline. Specific evidence indicates that aerobic exercise combined with dietary intervention can improve cognitive function and daily living independence in postmenopausal obese women [23]. In elderly individuals with subjective cognitive impairment, multimodal interventions (cognitive training, diet, exercise) are superior to cognitive training alone [24,28]. Additionally, long-term MD combined with exercise intervention can help alleviate hippocampal atrophy and lateral ventricular dilation in middle-aged and elderly populations, resulting in higher overall cognitive scores and a lower risk of cognitive decline [29–31]. Research indicates that comprehensive lifestyle interventions aimed at enhancing adherence to the MD and increasing PA can significantly improve dietary behaviors in older adults, with both individual and combined approaches demonstrating feasibility [27]. Furthermore, such combined interventions may exert independent and synergistic effects on cognitive function. Adherence to an MD combined with an exercise program shows promise for improving spatial working memory and emotional well-being in older adults [30]. Although long-term intervention studies are challenging due to the progressive nature of cognitive decline, combined interventions retain distinct advantages [66]. For instance, individuals with high adherence to both the MD and high PA levels demonstrate a lower risk of developing AD [67]. One-year follow-up data from one trial revealed that participants originally assigned to a combined aerobic exercise and MD intervention maintained optimal executive function, followed by the diet-only group; both groups significantly outperformed the control group [68]. However, the complexity of such trials, with numerous uncontrollable factors and a scarcity of experimental research, presents challenges for future studies. The confounding effects of other dietary patterns also warrant attention. One prospective study found that high consumption of Western dietary components counteracted the cognitive benefits of the MD over a 6.3-year follow-up period [69]. Only when Western dietary intake was low did high adherence to the MD significantly delay cognitive decline. In a prospective study of 323 Scottish elderly participants, lower adherence to the MD was associated with greater overall brain volume loss over a 3-year period [70]. Research has found that higher adherence to the MD is significantly associated with preserved white matter microstructure and enhanced structural connectivity [71]. Therefore, adherence to the MD is one of the key behavioral factors in protecting cognitive function.

Summary, older adults should prioritize a Mediterranean dietary pattern, with a focus on increasing intake of nuts, fish, berries, leafy green vegetables, and olive oil, while reducing red meat and processed foods. Regarding physical activity, achieving at least 150 min of moderate-intensity aerobic exercise per week (e.g. brisk walking, Tai Chi) is a reasonable goal; however, the critical influence of exercise dosage and individual compliance on outcomes must be considered. Increasing the intake of vitamin D‑rich foods and engaging in brief outdoor activities around midday can also contribute to cognitive protection when sunlight exposure is insufficient. Multi‑modal intervention (diet, exercise, and cognitive training) is most effective for individuals with subjective cognitive impairment or those at high risk, whereas early intervention (e.g. around age 50) may be more effective in delaying brain atrophy and cognitive decline. Communities and medical institutions should promote low‑cost, scalable lifestyle intervention programs and incorporate dietary and exercise assessments into routine health management for older adults to effectively reduce the risk of dementia and related cognitive impairments. It is worth noting that implementing a Mediterranean diet and exercise interventions has indeed imposed a burden on the older adults, including physical fatigue, psychological pressure associated with long-term adherence, and the costs of lifestyle adjustment. However, this burden is not insurmountable. We further emphasize that clinicians' active recommendations and full support play a key role in mitigating this burden. When clinicians provide personalized recommendations based on individual health status (e.g. comorbidities, physical fitness level, cognitive function), along with continuous monitoring and encouragement, they can significantly improve the older adults' self-efficacy and adherence. Therefore, we call on clinicians to translate evidence-based dietary and exercise strategies into acceptable and sustainable daily practices for the older adults through regular follow-up, family involvement, and flexible adjustment protocols.

### 4.2. Mechanisms underlying the effects of a Mediterranean diet combined with physical activity intervention on cognitive function in older adults

#### 4.2.1. Protective mechanisms of the Mediterranean diet in enhancing cognitive function in older adults

The MD, widely regarded as a healthy dietary pattern, exhibits neuroprotective properties [22]. Research indicates that greater adherence to this diet among older adults is strongly associated with higher cognitive scores and is linked to a 30%–40% reduction in the risk of AD [72]. The core protective mechanisms of the diet primarily involve the anti-inflammatory and antioxidant effects of its components, as well as the optimization of cerebrovascular function.

Foods characteristic of the MD confer anti-inflammatory benefits through multi-targeted mechanisms, including the suppression of pro-inflammatory cytokines and the promotion of anti-inflammatory ones. These effects contribute to reduced levels of AD-related proteins such as amyloid-β, tau, and NfL, along with decreased inflammatory markers including CRP, IL-6, and TNF-α [22]. For instance, omega-3 fatty acids—found in deep-sea fish and walnuts—mitigate excessive microglial activation by suppressing the NF-κB pathway, which in turn reduces pro-inflammatory factors such as IL-6 and TNF-α [37]. They also inhibit the activity of cyclooxygenase-2 and nitric oxide synthase-2, thereby improving cognitive function in AD patients [37]. Similarly, oleocanthal in olive oil possesses cyclooxygenase-inhibiting properties comparable to ibuprofen, directly blocking neuroinflammatory pathways. Polyphenols from sources like red wine and berries modulate Treg cell function to maintain immune homeostasis and reduce β-amyloid-induced inflammatory responses [36].

The MD activates antioxidant defense systems. Bioactive components of this dietary pattern—such as antioxidant vitamins, polyphenols, other phytochemicals, and unsaturated fatty acids—improve neurogenesis, synaptic plasticity, and neuronal survival by mitigating oxidative stress (OS) [73]. As plant-derived polyphenolic compounds, flavonoids have been shown in studies to potentially alleviate cognitive impairment. They exert neuroprotective effects primarily through mechanisms such as reducing neuroinflammation, lowering OS, and promoting neuroplasticity [27,74]. Similarly, resveratrol from grape skins activates the longevity protein SIRT1, which enhances mitochondrial function and reduces OS [75]. Collectively, these mechanisms are linked to cognitive processes critical for memory and executive function [76].

The MD is associated with optimized brain structure and function. Research on brain health indicates that this dietary pattern significantly strengthens structural connectivity within the left hemisphere, particularly in regions including the amygdala, lingual gyrus, olfactory cortex, central occipital gyrus, and calcarine gyrus [50]. Greater adherence to the MD correlates with reduced mean diffusivity and increased fractional anisotropy in white matter, reflecting enhanced microstructural integrity. Furthermore, the diet's components confer specific cerebrovascular benefits. Monounsaturated fatty acids from olive oil upregulate endothelial nitric oxide synthase (eNOS) activity, thereby improving cerebral perfusion [23]. Dietary nitrates, found in spinach and beetroot, promote vasodilation and increase hippocampal oxygen supply [77]. The MD also elevates high-density lipoprotein cholesterol (HDL-C) levels, which mitigates the risk of cerebral microinfarction by reducing atherosclerosis [78].

#### 4.2.2. The protective mechanisms of physical activity on cognitive function in older adults

The acceleration of global population ageing has established cognitive decline in older adults as a critical public health issue. Physical activity confers multifaceted benefits on cognitive function through diverse physiological and psychological mechanisms [79]. These benefits are not limited to delaying cognitive decline but may also include the partial reversal of certain functional impairments [80]. Consequently, regular PA effectively enhances cognitive capacity and can slow the progression of neurodegenerative diseases, such as AD and Parkinson's disease [81].

Physical activity enhances the efficiency of neural network activation. In older adults, this enhancement can mitigate cognitive decline through mechanisms of neural compensation and resource reorganization. Research demonstrates that exercise significantly improves performance on logical memory tests, indicating a close association between exercise-related network activation and cognitive function [82]. Notably, PA may bolster cognitive reserve (CR) not primarily by increasing the intensity of neural activation, but by reducing neural load and improving neural network efficiency [52]. Supporting this, a three-month PA intervention improved semantic memory across elderly individuals with varying cognitive status; paradoxically, functional magnetic resonance imaging (fMRI) detected decreased activation levels in the corresponding brain regions [83].

Physical activity influences the expression of neurotransmitters. Research demonstrates that exercise increases the bioavailability of neurotrophic factors, including BDNF, insulin-like growth factor-1 (IGF-1), and vascular endothelial growth factor (VEGF). This enhancement regulates synaptic plasticity and improves cognitive function [84–86]. During exercise, BDNF crosses the blood-brain barrier. Proteins such as cathepsin B and FNDC5, along with their cleavage products (e.g. irisin) and metabolites secreted by peripheral muscles, also cross this barrier. This process facilitates BDNF expression in the hippocampus, ultimately promoting neurogenesis and improving memory function [87–89]. IGF-1 promotes neuronal growth, survival, and differentiation [86]. Elevated cerebral IGF-1 levels support neuronal proliferation, survival, and plasticity, thereby providing neuroprotection and stimulating angiogenesis [90]. In older adults, various exercise regimens can increase levels of both IGF-1 and noradrenaline, with the latter showing a significant correlation with working memory [91]. Furthermore, exercise elevates VEGF levels, optimizes vascular velocity, and consequently improves vascular function [92]. Finally, exercise augments prefrontal cortex function through neurotransmitter systems such as dopamine and serotonin, which strengthen executive control abilities [93].

Physical activity exerts anti-inflammatory and antioxidant effects, which can help counteract pathways leading to neurodegeneration. OS, induced by reactive oxygen species (ROS), promotes amyloid-β (Aβ) deposition and the aggregation of hyperphosphorylated tau proteins. Regular exercise reduces levels of pro-inflammatory factors, including interleukin-6 (IL-6) and tumour necrosis factor-alpha (TNF-α), thereby mitigating associated neuronal damage [94]. For instance, low-intensity exercises such as Tai Chi have been shown to significantly enhance superoxide dismutase (SOD) activity. Furthermore, exercise increases the inhibition of FNDC5, which suppresses the expression of inflammatory cytokines in the brain and improves cognitive impairment [88]. Aerobic exercise also upregulates the expression of BDNF [80]. This enhancement is coupled with an increase in the anti-apoptotic factor B-lymphoma cell-derived factor (B-LDF) and a reduction in related pro-apoptotic factors, effectively inhibiting neuronal apoptosis and delaying brain ageing [95]. Finally, exercise-generated exosomes suppress OS-induced neuronal apoptosis and significantly enhance neuronal viability [94].

Physical activity optimizes the brain's microenvironment through several mechanisms. First, exercise enhances cerebrovascular function. Physical exertion stimulates vascular endothelial cells to release nitric oxide, which dilates cerebral blood vessels and increases cerebral blood flow (CBF) [80]. This process not only increases grey matter volume in the frontal lobe and hippocampus but also promotes the secretion of neurotransmitters, including serotonin and noradrenaline, and upregulates neurotrophic factors, thereby enhancing neuroplasticity and optimizing synaptic connections [80]. Concurrently, exercise improves the integrity and function of cerebral blood vessels and enhances the efficiency of glucose and lipid metabolism, ensuring a greater supply of oxygen and nutrients to the brain [96]. Furthermore, exercise modulates VEGF levels, inducing alterations in cerebral blood flow that are crucial for maintaining the blood-brain barrier [97]. These molecular-level changes collectively contribute to improved cognitive function. Second, exercise facilitates the clearance of metabolic waste. It accelerates the removal of byproducts such as tau protein via the lymphatic system, a process that is significantly enhanced during sleep [98]. Regular aerobic and resistance training also effectively counteract oxidative damage, neuroinflammation, and amyloid-beta deposition, while promoting neurogenesis, synaptic plasticity, cerebral blood flow perfusion, and mitochondrial function [62]. This leads to the inhibition of neuroinflammation, a reduction in OS, and enhanced cognitive performance [99]. For example, exercise can intervene in Alzheimer's disease-related pathological processes through multiple pathways, including regulating β-amyloid (Aβ) metabolism, reducing neuroinflammation, promoting the synthesis and release of neurotrophic factors, and improving cerebral blood flow (CBF) [100]. Notably, exercise can induce the generation of exosomes [101], which further facilitate the clearance of metabolic waste products like amyloid-beta [94].

#### 4.2.3. Synergistic mechanisms of the Mediterranean diet combined with physical activity intervention on cognitive function in older adults

The MD and PA are two distinct health interventions widely recognized for delaying cognitive decline in older adults. The MD, which emphasizes olive oil, fish, nuts, whole grains, and fresh fruits and vegetables, is rich in omega-3 fatty acids, polyphenols, and antioxidants. These components possess antioxidant and anti-inflammatory properties, which are beneficially associated with a reduction in age-related neurodegenerative diseases. These bioactive compounds can cross the blood-brain barrier [102], where they suppress neuroinflammation, mitigate brain ageing caused by free radical damage and inflammatory cytokines, and promote hippocampal cell proliferation and adult neurogenesis, thereby enhancing cognitive function [103]. Regular PA, particularly aerobic activity and resistance training, stimulates the secretion of BDNF, enhances hippocampal neurogenesis, and improves cerebral blood flow perfusion [84–86]. When these two interventions act in synergy, their combined effect can be greater than the sum of their individual parts (a synergistic phenomenon). Research indicates that older adults who adhere to both the MD and regular weekly exercise demonstrate greater cognitive improvement than those who receive only a single intervention [30,68].

At the molecular level, a positive feedback loop exists between the omega-3 fatty acids central to the MD, such as docosahexaenoic acid (DHA), and exercise-induced BDNF secretion. DHA, a primary structural component of neuronal membranes [102], promotes BDNF gene expression by activating the PPAR-γ pathway [87]. In turn, BDNF enhances the neuronal uptake efficiency of DHA. Concurrently, polyphenolic compounds like oleuropein in olive oil mitigate transient post-exercise inflammation by inhibiting the NF-κB pathway [73,103], thereby protecting exercise-induced neuroplasticity [7]. At the cellular level, this synergy optimizes mitochondrial function through complementary mechanisms: dietary antioxidants (e.g. vitamin E) scavenge free radicals [74], while exercise promotes mitochondrial biogenesis via the AMPK pathway, together improving neuronal energy metabolism [94]. Notably, the 30-min “nutritional window” post-exercise is critical for these synergistic effects [29]. Consuming nuts or olive oil during this period prolongs the BDNF peak by 2.1 h and increases the expression of synaptic plasticity-related proteins, such as PSD-95, by 40% [104].

**Figure 2.**
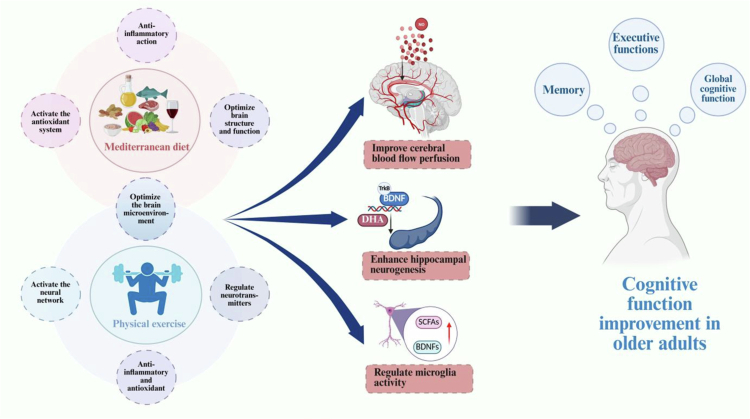
Mechanisms underlying the cognitive benefits of combining the Mediterranean diet with physical exercise in older adults.

From a systemic perspective, the MD and PA exhibit synergistic effects in maintaining brain health through multiple physiological pathways. Regarding vascular function, dietary monounsaturated fatty acids work in concert with exercise-induced nitric oxide (NO) production. Specifically, the oleic acid in olive oil upregulates endothelial nitric oxide synthase (eNOS) expression to enhance vasodilation [105], while exercise further activates this pathway through mechanical stimulation, substantially increasing cerebral blood flow [92,106]. This vascular optimization is critical for preventing vascular cognitive impairment by ensuring adequate neuronal supply of oxygen and nutrients. In terms of brain structure, exercise-promoted BDNF secretion and dietary omega-3 fatty acids act synergistically on the hippocampus: BDNF stimulates neurogenesis [62], and docosahexaenoic acid (DHA) enhances the survival of new neurons by activating the CREB pathway, thereby reducing annual hippocampal atrophy [46]. Notably, both interventions establish cross-protective mechanisms via the gut–brain axis. Short-chain fatty acids (SCFAs), produced by gut microbiota from dietary fiber, cross the blood-brain barrier and collaborate with exercise-induced BDNF to modulate microglial activity, reducing levels of the neuroinflammatory marker IL-6 [106,107]. Ultimately, this multisystemic synergy enhances overall cognitive function, including episodic memory, executive function, and information processing speed [28,31,68]. Therefore, the beneficial effects of the combined MD and exercise intervention are achieved through multidimensional pathways. The diet provides the essential nutritional substrate, while exercise activates neuroplasticity pathways, thereby establishing a dual defense for brain health. The MD and PA exert their synergistic effects primarily at the molecular and systemic levels (Figure 2).

## 5. Limitations

This review has several limitations. First, the scarcity of high-quality experimental studies precludes the conduct of meta-analyses or structured systematic reviews. Only 7 studies were included. A small number of studies led to insufficient statistical grasp, increased risk of publication bias, and limited the extrapolation of conclusions. In the future, more large samples and high-quality studies are needed to verify. Most studies included in the review relied on dietary questionnaires to assess adherence and lacked validation with biomarkers; the dose-response relationship of exercise interventions also remains unclear. Moreover, few studies implemented a tripartite intervention strategy integrating diet, exercise, and cognition. Future research could explore such integrated approaches, For instance, by combining nutritional interventions with PA and monitoring the gut-brain axis, while ensuring nutritional balance. Finally, tailoring dietary and exercise programs to regional cultural characteristics can enhance their applicability and effectiveness in promoting senior health. However, the modified programs must still align with medical dietary principles.

## 6. Conclusions

Maintaining optimal nutritional and metabolic health is critical for supporting cognitive function in older adults. The Mediterranean diet, a pattern rich in polyunsaturated fatty acids, antioxidants, and anti-inflammatory compounds, helps preserve cognitive abilities and delay decline. Regular physical activity also significantly mitigates cognitive deterioration by promoting neurotrophic factor release, enhancing synaptic plasticity, increasing cerebral blood flow, and facilitating neurotransmitter activity. Individual adherence to both the Mediterranean diet and an exercise regimen constitutes a key behavioral determinant for sustaining cognitive health. Importantly, these two lifestyle components interact synergistically, creating a virtuous cycle that enhances brain plasticity and overall cerebral resilience. Based on current evidence, a combined intervention of the Mediterranean diet and regular physical activity is recommended to protect cognitive function, ideally implemented during the prodromal stages before pathological cascades become irreversible.

## Data Availability

According to legitimate requirements, goods can be sold to the corresponding author.
